# Structural and functional alterations in heart and skeletal muscle following severe TAC in mice: impact of moderate exercise training

**DOI:** 10.1186/s40659-021-00354-2

**Published:** 2021-09-19

**Authors:** Julia Böttner, Sarah Werner, Volker Adams, Sarah Knauth, Angela Kricke, Holger Thiele, Petra Büttner

**Affiliations:** 1 Department of Cardiology, Heart Center Leipzig at University of Leipzig, Strümpellstr. 39, 04289 Leipzig, Germany; 2 Laboratory of Molecular and Experimental Cardiology, TU Dresden, Heart Center Dresden, Dresden, Germany

**Keywords:** Heart failure, Transverse aortic constriction, Moderate exercise, Intervention, Treadmill, Peripheral cachexia, Force production

## Abstract

**Background:**

Heart failure (HF) is the leading cause of death in western countries. Cardiac dysfunction is accompanied by skeletal alterations resulting in muscle weakness and fatigue. Exercise is an accepted interventional approach correcting cardiac and skeletal dysfunction, thereby improving mortality, re-hospitalization and quality of life. Animal models are used to characterize underpinning mechanisms. Transverse aortic constriction (TAC) results in cardiac pressure overload and finally HF. Whether exercise training improves cardiac remodeling and peripheral cachexia in the TAC mouse model was not analyzed yet. In this study, 2 weeks post TAC animals were randomized into two groups either performing a moderate exercise program (five times per week at 60% VO_2_ max for 40 min for a total of 8 weeks) or staying sedentary.

**Results:**

In both TAC groups HF characteristics reduced ejection fraction (− 15% compared to sham, p < 0.001), cardiac remodeling (+ 22.5% cardiomyocyte cross sectional area compared to sham; p < 0.001) and coronary artery congestion (+ 34% diameter compared to sham; p = 0.008) were observed. Unexpectedly, peripheral cachexia was not detected. Furthermore, compared to sedentary group animals from the exercise group showed aggravated HF symptoms [heart area + 9% (p = 0.026), heart circumference + 7% (p = 0.002), right ventricular wall thickness − 30% (p = 0.003)] while muscle parameters were unchanged [Musculus soleus fiber diameter (p = 0.55), Musculus extensor digitorum longus contraction force (p = 0.90)].

**Conclusion:**

The severe TAC model is inappropriate to study moderate exercise effects in HF with respect to cardiac and skeletal muscle improvements. Further, the phenotype induced by different TAC procedures should be well documented and taken into account when planning experiments.

## Introduction

Approximately 64.3 million people worldwide suffer from heart failure (HF). In European countries the prevalence is up to 1.3–4% [1]. Despite continuously improving therapies in HF the 5- and 10-year mortality still is estimated to be 53% and 65%, respectively [2]. Two thirds of HF patients suffer from skeletal muscle atrophy [3], fatty infiltration, decreased antioxidant capacity and show muscle fiber type shift from slow-twitching to fast-twitching muscle fibers resulting in a metabolic shift from aerobic, oxidative to a rather anaerobic, glycolytic metabolism [4]. These molecular alterations in skeletal muscle structure and composition result in a loss of lean body mass, muscle weakness and fatigue [3]. Nevertheless, HF can be prevented or decelerated by modifying behavioral patterns. One easy-to-address and establish cardioprotective habit is physical activity, because it has the ability to correct the structural and metabolic alterations in the peripheral muscle [4–8]. Regular exercise is beneficial for cardiac health by reducing risk factors [6, 9]. Furthermore, exercise improves quality of life in HF patients by increasing exercise tolerance and oxygen uptake [7, 10, 11]. Exercise-based cardiac training is known to effectively reduce total and cardiovascular mortality as well as hospital admission [12].

Despite the accepted protective effect of exercise on HF, the molecular basis is still not completely understood. This is hindering the development of individualized exercise regimens and the identification of underpinning regulatory pathways that may be targeted pharmaceutically.

Different animal models, that imitate human pathologies, are accessible for HF induction. In patients with HF due to myocardial infarction exercise improves cardiac remodeling and function [13]. Thus, ligation of the left anterior descending artery (LAD) is a common method to induce myocardial infarction and subsequently HF in animal models. A drawback of this model is high mortality and low reproducibility [14].

To overcome these obstacles transverse aortic constriction (TAC) is used to induce HF whereas low mortality and high reproducibility are reported for this method [15]. TAC limits the left ventricular (LV) outflow resulting in LV pressure overload and fast evolving HF characterized by cardiac hypertrophy and remodeling as well as decreasing cardiovascular performance [16, 17]. To standardize the procedure a needle is placed next to the aorta, a full constriction is placed and after the needle is removed, the final aortic diameter is determined. Lower needle diameters determine more severe hypertrophy as well as accelerated time course of HF development [15, 16]. Mild TAC introduced by aortic banding using a 25 gauge (G) needle represents a mild model of hypertrophy and diastolic dysfunction without systolic dysfunction or cardiac fibrosis, consistent with the human condition of hypertensive heart disease [16]. Moderate 26G TAC results in systolic dysfunction with perivascular cardiac fibrosis. Severe TAC applied by using a 27G needle eventuates in systolic and diastolic dysfunction, distinct cardiac fibrosis, hypertrophy and secondary increased lung mass, consistent with pulmonary edema [16].

The effect of exercise on HF development and associated muscle remodeling using the TAC model in mice was studied by Wang et al. and Tian et al. [8, 18]. They observed improved cardiac performance following exercise training with increased intensity [8]. Most existing studies on TAC in combination with exercise intervention focus on cardiac effects and lack information about TAC- and exercise-induced structural, functional and metabolic alterations of the skeletal muscles. On that account, we applied severe cardiac pressure overload by banding the aorta using a 27G needle to analyze the effects of moderate training in HF on cardiac and skeletal muscle structure and function. To our best knowledge, this is the first study to address both, cardiac and skeletal muscle performance in HF mice after exercise intervention.

## Results

### Evaluation of TAC phenotype

In the 48 h post-operative period six mice died following 27G severe TAC, two mice died following sham surgery. In TAC_SED_ one mouse died 22 d post-surgery from unknown cause, which led to final groups of n = 13 in sham, n = 11 in sedentary (TAC_SED_) and n = 12 in exercising TAC (TAC_EX_) groups.

**Fig. 1:**
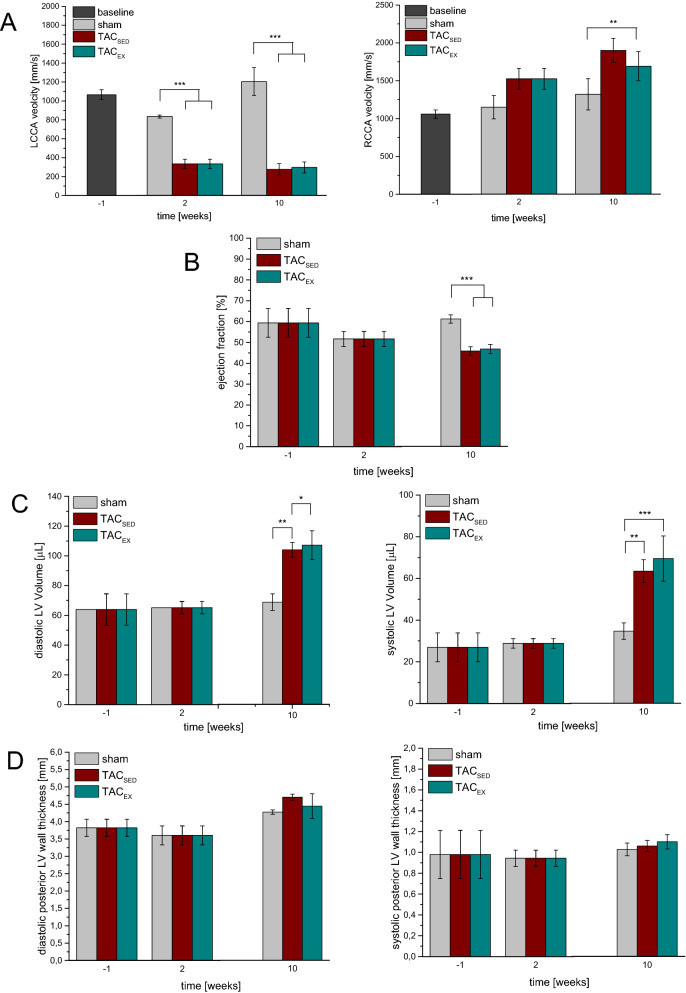
Cardiac characteristics 1 week before TAC surgery (− 1), 2 weeks post TAC surgery (2), and 10 weeks post TAC whereas TAC_EX_ animals did 8 weeks of moderate training. Quantification of the left (LCCA, left graph) and right common carotid (RCCA, right graph) velocity (**A**), ejection fraction (**B**) and left ventricular (LV) volume (**C**) and posterior wall thickness (**D**) during diastolic (left) and systolic (right) phase using echocardiography. Mean ± SEM. *n* ≥ 11. Two-way ANOVA with Bonferroni correction. *p < 0.05, **p < 0.01, ***p < 0.001, vs. sham

Echocardiographic measurements (Fig. 1A) confirmed establishment of TAC 2 weeks after surgery. The velocity in the left common carotid artery (LCCA) decreased after TAC (p < 0.001), whereas the right common carotid artery (RCCA) velocity only numerically increased after TAC compared to sham-operated animals (p = 0.098).

Ten weeks post-surgery body weights (BW) and tibial length (TL) in TAC_SED_ and TAC_EX_ were comparable to sham. All results are summarized in Table 1.

**Table 1:** Functional and morphological characteristics of sham, TAC_SED_ and TAC_EX_

	Sham	TAC_SED_	TAC_EX_
Baseline TAC phenotype			
Body weight (g)	28.2 ± 0.1	28.2 ± 0.2 (*p* = 0.13)	29.2 ± 0.1 (a: *p* = 0.95; b: *p* = 0.18)
Tibia length (mm)	17.2 ± 0.05	17.5 ± 0.03 (*p* = 0.89)	17.2 ± 0.05 (a: *p* = 0.10; b: *p* = 0.12)
Heart weight (mg)	145.6 ± 1.0	201.4 ± 2.8 (*p* < 0.001)	213.8 ± 2.8 (a: *p* < 0.001; b: *p* = 0.46)
Heart weight/tibia length (mg/mm)	8.5 ± 0.1	11.6 ± 0.1 (*p* < 0.001)	12.4 ± 0.2 (a: *p* < 0.001; b: *p* = 0.26)
EDL mass/TL (mg/mm)	0.77 ± 0.12	0.83 ± 0.05 (*p* = 0.14)	0.81 ± 0.05 (a: *p* = 0.35; b: *p* = 0.61)
Functional cardiac parameters			
LCCA velocity 2 weeks post TAC (mm/s)	833.5 ± 15.3	333.0 ± 49.5 (a: *p* < 0.001; b: *p* = 0.90)	
RCCA velocity 2 weeks post TAC (mm/s)	1148.7 ± 154.4	1524.1 ± 138.0 (a: *p* = 0.098; b: *p* = 0.42)	
LVEF (%)	61 ± 5	46 ± 6 (*p* < 0.001)	47 ± 7 (a: *p* < 0.001; b: *p* = 0.76)
FS (%)	32 ± 3	22 ± 4 (*p* < 0.001)	23 ± 4 (a: *p* < 0.001; b: *p* = 0.76)
Diastolic LV volume (µL)	68.7 ± 5.58	103.9 ± 4.9 (p = 0.002)	107.1 ± 9.6 (a: *p* = 0.03; b: *p* = 0.77)
Systolic LV volume (µL)	34.6 ± 4.0	63.4 ± 5.5 (*p* < 0.001)	69.4 ± 10.9 (a: *p* = 0.007; b: *p* = 0.63)
Diastolic LV wall thickness (mm)	4.3 ± 0.1	4.7 ± 0.9 (*p* = 0.27)	4.4 ± 0.4 (a: *p* = 0.097; b: p = 0.69)
Systolic LV wall thickness (mm)	1.0 ± 0.1	1.1 ± 0.1 (*p* = 0.64)	1.1 ± 0.1 (a: p = 0.50; b: *p* = 0.82)
Histological evaluation of cardiac remodeling			
Relative heart area	1	0.98 ± 0.05 (*p* = 0.72)	1.09 ± 0.03 (a: *p* = 0.022; b: *p* = 0.053)
Relative circumference	1	0.98 ± 0.02 (*p* = 0.45)	1.07 ± 0.02 (a: *p* = 0.01; b: *p* = 0.003)
Relative LV thickness	1	1.09 ± 0.08 (*p* = 0.40)	1.09 ± 0.06 (a: *p* = 0.23; b:*p* = 0.97)
Relative RV thickness	1	0.77 ± 0.08 (*p* = 0.044)	0.70 ± 0.06 (a:*p* = 0.004; b: *p* = 0.56)
Cardiomyocyte CSA (µm^2^)	50.3 ± 0.8	61.6 ± 1.2 (*p* < 0.001)	59.6 ± 1.0 (a: *p* < 0.001; b: *p* = 0.20)
Perivascular fibrosis (%)	1.8 ± 0.1	2.0 ± 1.1 (*p* = 0.75)	2.6 ± 0.2 (a:*p* = 0.27; b:*p* = 0.48)
Interstitial fibrosis (%)	0.2 ± 0.1	0.4 ± 0.1 (*p* = 0.51)	0.3 ± 0.1 (a: p = 0.01; b: p = 0.86)
Coronary artery diameter (µm)	27.3 ± 2.0	36.6 ± 2.8 (*p* = 0.008)	30.8 ± 3.0 (a: *p* = 0.32; b: *p* = 0.16)
Muscle function and remodeling			
EDL max. force (N/cm^2^)	25.8 ± 5.7	28.8 ± 4.7 (*p* = 0.20)	28.1 ± 6.6 (a: *p* = 0.30; b: *p* = 0.9)
EDL fiber diameter (µm)	40.7 ± 1.7	35.3 ± 1.6 (*p* = 0.04)	37.1 ± 1.4 (a: *p* = 0.16, b: *p* = 0.79)
EDL slow fiber proportion (%)	2.25 ± 0.41	1.81 ± 0.48 (*p* = 0.78)	2.63 ± 1.12 (a: *p* = 0.62; b: *p* = 0.53)
EDL fast fiber proportion (%)	92.7 ± 1.1	86.6 ± 07.2 (*p* = 0.32)	93.1 ± 4.6 (a: *p* = 0.84; b: *p* = 021)
Soleus fiber diameter (µm)	44.3 ± 1.9	45.8 ± 1.5 (*p* = 0.74)	37.8 ± 1.5 (a: *p* = 0.01; b: *p* = 0.002)
Soleus slow fiber proportion (%)	30.3 ± 3.0	33.6 ± 8.5 (*p* = 0.73)	29.7 ± 2.6 (a: *p* = 0.87; b: *p* = 0.78)

Mean ± SEM (Two-Way ANOVA with Bonferroni correction, a: *p* value TAC_EX_ vs. sham, b: *p* value TAC_SED_ vs. TAC_EX_, a and b are listed in the fourth column “TAC_EX_”)

*EDL* extensor digitorum longus, *LVEF* left ventricular ejection fraction, *FS* fractional shortening, *RV* right ventricle, *CSA* cross-sectional area

### Severe TAC alters cardiac structure and function in sedentary animals

**Fig. 2:**
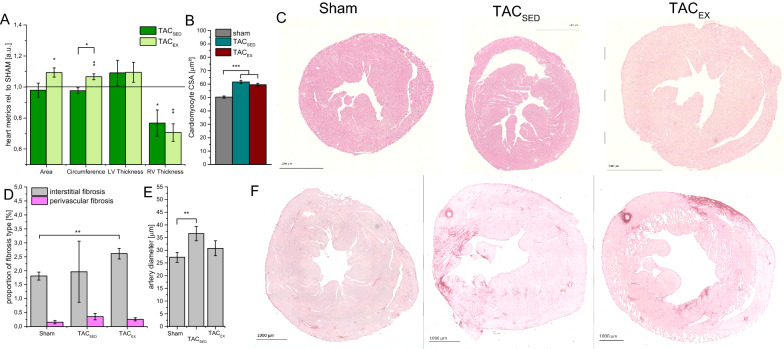
Effects of pressure overload and moderate training on cardiac hypertrophy and fibrosis. **A** Calculated area, circumference, left ventricular (LV) and right ventricular (RV) wall thickness of TAC-operated mice in relation to sham-operated control, **B** cardiomyocyte cross-sectional area (CSA), **C** hematoxylin/eosin stained heart cross-sections. **D** Quantification of the TAC-induced fibrosis and **E** coronary artery diameter using **F** Sirius-Red stained heart cross-sections. Mean ± SEM, *n* ≥ 5. One-way ANOVA with Bonferroni correction. *p < 0.05, **p < 0.01, ***p < 0.001

Cardiac remodeling after TAC operation was evaluated by analysis of heart metrics, development of interstitial and perivascular fibrosis as well as coronary artery remodeling 10 weeks post TAC (see Fig. 2 and Table 1).

The area and circumference in TAC_SED_ were comparable to the sham operated control group. LV thickness of heart cross sections was slightly decreased in TAC_SED_. Interestingly, the right ventricular (RV) wall thickness in TAC_SED_ was significantly reduced by 22% in comparison to the sham group (p = 0.049 see Fig. 2A). Cardiomyocyte cross sectional area (CSA) in TAC_SED_ was significantly increased by 22% in contrast to sham (see Fig. 2B). The amount of interstitial and perivascular fibrosis in TAC_SED_ was comparable to sham (see Fig. 2D). The coronary artery diameter in TAC_SED_ was significantly increased by 9 µm (p = 0.008 vs. sham; Fig. 2E). The heart weight of the TAC_SED_ was increased up to 1.4-fold in contrast to sham. Echocardiographic analyses revealed an increase in diastolic LV volume by 50% (p = 0.002), but no significant alterations in LV posterior wall thickness (p = 0.27) as well as a significant reduction in left ventricular ejection fraction (LVEF) of 15% compared to sham in TAC_SED_. Additionally, systolic LV volume was significantly increased by 28 µL (p < 0.001), although LV wall thickness in the systolic phase was unaltered in TAC_SED_ (p = 0.64).

### Exercise effects on TAC induced cardiac dysfunction

We analyzed the impact of moderate exercise training (TAC_EX_) on the cardiac alterations observed in TAC_SED_ (Fig. 2 and Table 1). Cardiac CSA was increased by 8% (p = 0.026) and heart circumference was increased by 6% (p = 0.002), while RV wall thickness was decreased by 30% (p = 0.003) in relation to sham. RV wall thickness reduction was observed in TAC_SED_ (23%) and TAC_EX_ (24%) without differences between these two groups (p = 0.56). In TAC_EX_ cardiomyocyte CSA was increased by 18% in contrast to sham (p < 0.001). In comparison to TAC_SED_, cardiomyocyte CSA remained similar in TAC_EX_ (p = 0.56). TAC_EX_ showed a 1.44-fold rise in cardiac interstitial fibrosis compared to sham (p = 0.01). No difference in the amount of interstitial fibrosis in TAC_SED_ and TAC_EX_ was observed. The amount of perivascular fibrosis in TAC_EX_ was comparable to sham and to TAC_SED_. Numerically, exercise intervention in the TAC operated group reduced the coronary artery diameter in the myocardium by 6 µm in TAC_EX_ compared to TAC_SED_. Heart weight in TAC_EX_ was significantly increased in contrast to sham (p < 0.001). No difference in heart weight between TAC_SED_ and TAC_EX_ was found. Functional cardiac parameters of TAC_EX_ are shown in Fig. 1. Diastolic and systolic LV volume in TAC_SED_ and in TAC_EX_ remained comparable. The diastolic and systolic LV wall thickness in TAC_SED_ were not improved in TAC_EX_. LV volume in diastolic and systolic phase remained 1.5- and twofold increased and the LVEF was reduced by 24% (p < 0.001) compared to sham. Additionally, no differences of LV output were found in TAC_SED_ and in TAC_EX_.

### Functional and structural alterations in skeletal muscle in sedentary and trained TAC mice

**Fig. 3:**
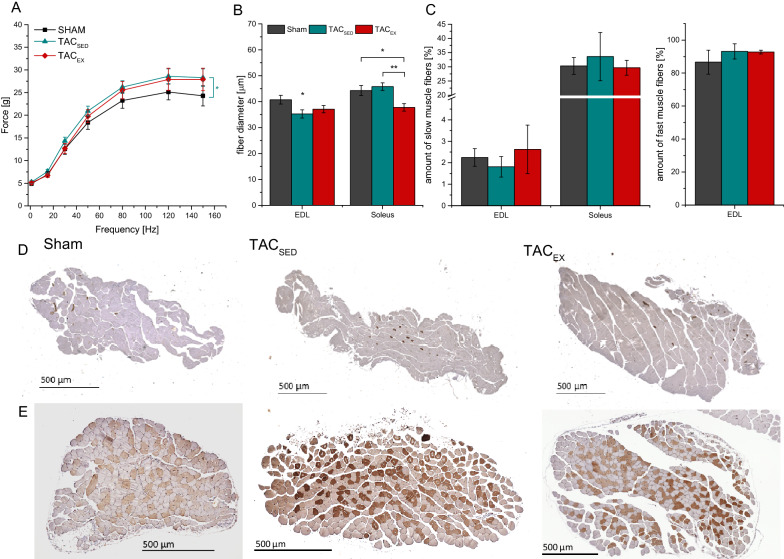
Effects of moderate training on muscle function of fast-twitching Extensor digitorum longus (EDL) and slow-twitching Soleus 10 weeks after TAC. In vitro absolute muscle forces of EDL (**A**) and fiber diameter (**B**). Proportion of slow and fast fibers (**C**) and representative images of slow muscle fiber staining in EDL (**D**) and Soleus (**E**). Mean ± SEM. Two-Way ANOVA with Bonferroni’s correction. *p < 0.05, **p < 0.01, ***p < 0.001

Effects of moderate exercise on HF were analyzed focusing on cardiac characteristics as well as peripheral muscle alterations. Extensor digitorum longus (EDL) contraction force, muscle fiber diameter and the proportion of slow-twitch and fast-twitch fibers in the EDL and the soleus muscle were analyzed to study the effects of moderate exercise in TAC induced HF 10 weeks after TAC surgery (see Table 1 and Fig. 3).

EDL contraction force was significantly increased in TAC_SED_ compared to sham (p = 0.017), but not in TAC_EX_. The EDL fiber diameter was significantly lower in TAC_SED_ (p = 0.044) but not in TAC_EX_ (p = 0.16), whereas there was no difference between both TAC groups.

Soleus muscle fiber diameter in TAC_SED_ remained comparable to sham. Interestingly, soleus muscle fiber diameter was significantly decreased (p = 0.002) in TAC_EX_ compared to sham. No alterations in the amount of slow and fast muscle fibers in EDL as well as in slow muscle fibers in soleus were observed.

## Discussion

We applied 27G severe TAC to induce a severe HF phenotype but chose a moderate exercise regimen to avoid overcharging TAC mice by exercise. Resulting cardiac dysfunction and peripheral cachexia as well as the potential capability of moderate exercise to improve them was examined. The results of the study can be summarized as follows:

1. additional aggravation of cardiac remodeling after exercise intervention
2. similar cardiac performance independently from exercise intervention
3. comparable structure and function of skeletal muscles in both, TAC_SED_ and TAC_EX_
4. EDL cross-sectional area was improved by exercise intervention, whereas the Soleus muscle showed further fiber atrophy following exercise intervention

### LV remodeling and dysfunction in severe TAC

In the present study 27G severe TAC induced HF characteristics in the mouse model. Increased diastolic and systolic LV filling accompanied by a significantly reduced EF were found, indicating LV dilatation and dysfunction as hallmarks of HF. The observed interstitial fibrotic and coronary artery remodeling is also in accordance to other studies [16, 19]. Cardiac remodeling in the TAC mouse model is accompanied by a congestion into coronary vessels [19] resulting in an increase in coronary artery diameter. We found an 1.34-fold increased coronary artery diameter in TAC_SED_ in comparison to sham, whereas Yang et al. found coronary artery diameter to be further increased by 2.3-fold [19].

In the current study no increase in perivascular fibrosis was observed while Richards et al. found increased perivascular fibrosis 4 weeks post 26G moderate TAC. Noteworthy, Richards et al. observed no interstitial fibrosis [16]. Yang et al. found interstitial and perivascular fibrosis 5 weeks after TAC in male mice (no needle diameter specified) [19]. Wang et al. [8] reported significant fibrosis 10 weeks after TAC (no needle diameter specified, presumably 27G [20]). Taken together, the development and pattern of fibrosis seems to depend strongly on the used needle diameter and the progressed time post TAC [7, 8]. Notably, our data suggest that the development of fibrosis in TAC_SED_ is highly individual, which is contrary to the concept that TAC results in an uniform and reproducible development of HF [15].

Based on the current results, future studies should include the specific TAC phenotype in their hypothesis generation including a detailed description of the performed TAC procedure as well as mouse strain, sex, age and origin. Furthermore, the high inter-individual variability in cardiac remodeling observed in this study should be taken into account when planning TAC experiments with regards to animal numbers and sample size calculation.

### RV remodeling in severe pressure overload

While we found no differences in LV wall thickness following TAC surgery, the RV wall was significantly thinner in both TAC groups. The echocardiographic analysis of the lateral RV wall thickness confirmed diastolic RV dilation, maybe resulting in RV dysfunction. RV dilatation as a consequence of LV pressure overload was addressed in only a few studies [21, 22] and thereby remains elusive. In pulmonary hypertension (PH) RV dilatation was found to represent an adjustment mechanism to reduce RV load [23]. Our data are in line with recent findings in TAC operated mice that showed distinct RV remodeling and diastolic dysfunction [22]. Platt et al. applied 26G TAC and monitored structural and functional RV and LV remodeling over 18 weeks. They postulated diastolic RV dysfunction to occur secondary to LV dysfunction. Finally, they showed establishment of PH secondary to RV dysfunction [22]. Interestingly, Platt et al. [22] found RV and LV peak pressure 4 weeks post TAC. While LV pressure normalized thereafter RV pressure remained elevated. This is well in line with our observations 10 weeks post TAC.

Right heart failure due to chronic RV overload is the major cause of death among patients with PH [24]. PH is associated with high mortality and its treatment is still challenging [25]. Furthermore, there is an unfortunate lack of sufficient animal models for PH. Therefore, we suggest the characterization of the TAC mouse model as a suitable model for PH type 2 [22], which is secondary to left heart disease [26].

### Result classification in the context of TAC induced HF and exercise intervention in mice

Numerous clinical and experimental studies showed that exercise improves cardiac structure and performance in ischemic cardiomyopathy [27–29], in systemic hypertension models [30, 31], in myocardial infarction [7] and reduces the rate of re-infarction and mortality in patients with HF [5]. Exercise-induced cardioprotection is underpinned by various molecular mechanisms, such as activation of heat shock and ER stress proteins as well as cyclooxygenase, enhancing the antioxidant capacity and autophagy processes [32]. Several studies investigated cardiac and skeletal muscle performance using mild to severe TAC with or without exercise in mice [8, 18]. Studies on TAC and exercise intervention always applied exercise as secondary intervention. Independently of the different starting times for exercise intervention (0–14 days post TAC) in the different studies, TAC was established at any time post-surgery, but no HF characteristics were verifiable at the exercise starting time.

Nine weeks of swimming exercise with an increasing duration were reported to induce beneficial cardiac effects in severe TAC operated mice [8]. LV mass was lower in comparison to TAC_SED_ while EF and FS were improved, indicating at least a partial recovery of cardiac output. Further, cardiomyocyte CSA, amount of fibrosis, catecholamines, nitric oxide and reactive oxygen species levels were improved in this study [8]. Correspondingly, improved LV mass, EF, FS, cardiomyocyte CSA, atrial and brain natriuretic peptide levels were reported following 8 weeks of treadmill running with increasing intensity in mice following severe TAC [18]. Due to these studies, we expected an improvement of HF-induced cardiac and skeletal muscle function following exercise training.

Instead, we observed more pronounced cardiac hypertrophy and thinning of the RV wall in TAC_EX_. Diastolic as well as systolic LV volume and wall thickness in TAC_EX_ were similar to TAC_SED_, indicating no beneficial effects of moderate exercise on cardiac remodeling or output. Exclusively, coronary artery remodeling induced by pressure overload may be at least partially reversed or prevented by exercise training.

Therefore, we comprehensively compared our data with the current literature on TAC-induced HF with a focus on exercise intervention or muscle function (Table 2). Van Deel et al. combined 25G mild TAC with 8 weeks of voluntary wheel run [6], which is better classified as a mild exercise training regime. Importantly, the TAC mice reduced their voluntary run distance indicating decreased wellbeing as observed in HF patients [6]. This is in strong contrast to the increasing intensity exercise regimes that were done by other groups [8, 18]. Van Deel et al. further observed an increase in LV mass in trained 25G TAC operated mice in contrast to sham. They showed a trending towards enhanced fibrosis following exercise training, accompanying the observed LV dysfunction [6], indicating that even voluntary exercise aggravates cardiac dysfunction in 25G mild TAC.

**Table 2:** Comparison of studies using the TAC model in C57/Bl6 mice with a focus on exercise intervention or muscle function in heart failure

PMID/author/year	Age at surgery, sex, animal supplier	Exercise start, exercise regime	TAC type	n	LV hypertrophy normalized to sham (%)	Cardiac characteristics at sacrifice	Muscle characteristics at sacrifice
28622359/Wang et al./2017	8 weeks, male, Jackson Lab	Start: 1 week post-surgery Regime: swimming with increasing training duration (20–60 min/day), 60% VO_2_max, 9 weeks	Sham sed	15	100	EF = 75%^a^, FS = 37%^a^	No muscle characteristics specified
			Sham ex	15	100^a^	EF = 80%^a^, FS = 40%^a^	No muscle characteristics specified
			TAC 27G sed	15	153	EF = 40%^a^. FS = 18%^a^. ~ 3-fold increased CSA, 12% interstitial fibrotic remodeling (Masson Trichrome staining)	No muscle characteristics specified
			TAC 27G ex	15	132	EF = 57%. FS = 25%. ~ 2-fold increased CSA. 4% interstitial fibrotic remodeling (Masson Trichrome staining)	No muscle characteristics specified
31930683/Tian et al./2019	6 weeks, male, Shanghai Lab Animal Center	Start: n.d Regime: Treadmill running with increasing intensity (30–60 min/day, 11–13 m/min), 8 weeks	Sham sed		100^a^	EF = 64%^a^, FS = 38%^a^	No muscle characteristics specified
			Sham ex		100^a^	EF = 70%^a^, FS = 40%^a^	No muscle characteristics specified
			TAC 27G sed		159^a^	EF = 50%^a^. FS = 23%^a^. ~ 1.4-fold increased CSA^a^, 2.8-fold increased ANP^a^, 3.5-fold increased BNP^a^	No muscle characteristics specified
			TAC 27G ex		140^a^	EF = 58%. FS = 30%. ~ 1.2-fold increased CSA^a^, twofold increased ANP^a^, 2.5-fold increased BNP^a^	No muscle characteristics specified
21291889/Van Deel et al./2011	20 weeks, sex not specified, supplier not specified	Start: immediately post-surgery Regime: 8 weeks voluntary wheel running	Sham sed	20	100	FS = 38%^a^	
			Sham ex	21	105^a^	FS = 40%^a^	Total run distance 432 ± 37 km
			TAC 25G sed	16	120^a^	FS = 32%^a^	
			TAC 25G ex	13	140	FS = 31%^a^	Total run distance 409 ± 42 km
			TAC 27G sed	35	186	FS = 17%^a^	
			TAC 27G ex	29	200^a^	FS = 14%^a^	Total run distance 276 ± 38 km
23449942/Richards et al./2013	8 weeks, male, Jackson Lab	ETT at 3, 7, 11, 15 weeks post-surgery	Sham	10	100	EF = 72 ± 4%	Maximum running distance at 3, 7, 11, 15 weeks: 630 m^a^, 410 m^a^, 390 m^a^, 400 m^a^
			LAD	34	147	EF = 36 ± 5%. Interstitial fibrotic remodeling (Masson Trichrome staining)	Maximum running distance at 3, 7, 11, 15 weeks: 490 m^a^, 380 m^a^, 400 m^a^, 390 m^a^
			TAC 27G	16	197	EF = 28 ± 6%. Interstitial fibrotic remodeling (Masson Trichrome staining)	Maximum running distance at 3, 7, 11, 15 weeks: 400 m^a^, 300 m^a^, 300 m^a^, 280 m^a^
29986381/Van Deel et al./2018	12–20 weeks, sex not specified, supplier not specified	Start: immediately post-surgery Regime: 8 weeks voluntary wheel running	Sham sed	12	100	FS = 38%^a^	
			Sham ex	12	100	FS = 40%^a^	Total run distance: 435 ± 6 km
			LAD sed	21	110^a^	FS = 10%^a^, collagen content ~ 1.8%^a^ increased	
			LAD ex	19	105^a^	FS = 15%^a^	Total run distance: 245 ± 5 km
			TAC sed, needle diameter not specified (presumably 27G)	19	200^a^	FS = 15%^a^, collagen content ~ 2% increased	
			TAC ex, needle diameter not specified (presumably 27G)	16	210^a^	FS = 13%^a^, collagen content ~ 3.7%^a^ increased	Total run distance: 237 ± 6 km
28159807/Sung et al./2017	8 weeks, male, Charles River Lab	ETT: treadmill running at 15 m/min, 3 and 5 weeks post TAC	Sham	15	100	EF = 50.08 ± 5.01%, FS = 25.73 ± 3.25% 5 weeks post TAC	Total run distance: 490 m^a^, whole body glucose consumption: 4.900 mL/kg/h^a^ and fatty acid oxidation: 600 mL/kg/h^a^, O_2_ consumption in EDL (a) basal: 2.1 nmol O_2_/min/mg dry wt^a^, (b) ADP-stimulated: 6.1 nmol O_2_/min/mg dry wt^a^ O_2_ consumption in Soleus (a) basal: 3 nmol O_2_/min/mg dry wt^a^, (b) ADP-stimulated: 2 nmol O_2_/min/mg dry wt^a^ All data 5 weeks post TAC
			TAC 27G	10	172	EF = 26.00 ± 3.47%, FS = 12.21 ± 1.78% 5 weeks post TAC	Total run distance: 260 m^a^, reduced activity in insulin pathway in skeletal muscle, whole body glucose consumption: 1.900 mL/kg/h^a^ and fatty acid oxidation: 800 mL/kg/h^a^, O_2_ consumption in EDL (a) basal: 1.3 nmol O_2_/min/mg dry wt^a^, (b) ADP-stimulated: 3 nmol O_2_/min/mg dry wt^a^ O_2_ consumption in Soleus (a) basal: 10 nmol O_2_/min/mg dry wt^a^, (b) ADP-stimulated: 4.9 nmol O_2_/min/mg dry wt^a^ All data 5 weeks post TAC
Own data	8 weeks, male, Janvier Lab	Start: 2 weeks post-surgery Regime: 8 weeks treadmill training at 60% VO_2_max	Sham	13	100	EF = 61 ± 5%, FS = 32 ± 3%	EDL maximum force 25.8 ± 5.7 N/cm^2^
			TAC_SED_ 27G	11	147	EF = 46 ± 6%, FS = 23 ± 4%, 1.2-fold increased CSA	EDL maximum force 28.8 ± 4.7 N/cm^2^ EDL: 1.2-fold decreased CSA, 1.25-fold decrease in slow fibers Soleus: CSA as in sham, 1.1-fold increase in slow fibers
			TAC_EX_ 27G	12	140	EF = 47 ± 7%, FS = 23 ± 4%, 1.2-fold increased CSA	EDL maximum force 28.1 ± 6.6 N/cm^2^ EDL: 1.1-fold decreased EDL CSA, 1.2-fold decrease in slow fibers Soleus: 1.2-fold decreased Soleus CSA, slow fibers as in sham

Left ventricular (LV) hypertrophy normalized to sham operated mice—simplified overview to allow for comparisons as some publications reported LV mass, LV mass/bodyweight, heart mass/tibial length or total heart weight (own data)

*n.d* not declared, *EF* ejection fraction, *FS* fractional shortening, *CSA* cross-sectional area, *ETT* exercise tolerance test, *ANP* Atrial Natriuretic Peptide, *BNP* Brain Natriuretic Peptide

^a^Value estimated from figure as not indicated otherwise

Richards as well as Gillis et al. characterized cardiac performance under moderate pressure overload applying 26G TAC [16, 33]. Both found higher LV mass and increased cardiomyocyte CSA four and up to 18 weeks post TAC [16], overall indicating a deterioration of cardiac performance in the course of time. Richards et al. performed exercise tolerance test at 3, 7, 11 and 15 weeks post 27G TAC and detected increasing exercise intolerance accompanied by decreasing EF in a time dependent aggravation [34]. Sung et al. observed decreased treadmill running distances 3 and 5 weeks post 27G severe TAC indicating exercise intolerance in HF mice [35]. They observed deteriorated LV mass, EF and FS [35]. Additionally, van Deel et al. examined the effects of mild exercise training on 27G severe TAC, observing a further increased LV mass as well as decreased FS down to 14% [6]. This is in accordance to our data, as we observed further aggravated cardiac performance following exercise intervention [6, 7, 34]. Neither LV mass nor EF, FS or fibrosis development were improved by exercise intervention, but rather additionally aggravated. Interestingly, Wang and Tian found intense exercise intervention to have beneficial effects on 27G severe TAC [8, 18], although van Deel et al. found mild exercise in 25G mild TAC to already deteriorate cardiac function [6]. The underlying cause for these contrasting results remain unclear. One limitation to the studies by Wang and Tian is that they didn’t test for maximum VO_2_ consumption before designing their exercise protocol [8, 18]. Instead they increased exercise intensity and duration without adapting the protocol according to individual capability of the HF mice in the first place. Wang et al. declare no mice were floating during swimming exercise [8], but did not discuss fear-evoking, psychological effects [36] of swimming exercise to the animals. Tian et al. did not mention, if mice dropped out during treadmill running [18]. We think careful observation of the HF mice during exercise training is essential, because progressing HF results in increased mice dropouts due to exhaustion. This is in accordance with EDL contraction force of TAC_EX_ showing no significant difference from Sham or TAC_SED_. One reason for this observation may be the severely diminished perfusion of the skeletal muscle due to increased peripheral vasoconstriction in HF. This in turn is caused by the impaired cardiac output [37].

One pitfall in the studies of Richards [34] and van Deel [6] is the lack of histological or molecular analyses of skeletal muscle alterations of TAC operated mice with and without exercise training. Our data show no beneficial influence of moderate exercise training on skeletal muscle structure or function. To our knowledge, the study by Sung et al. is the only one to assess metabolic alterations in skeletal muscles 5 weeks post TAC. They found a reduced activity in the insulin pathway in skeletal muscles resulting in reduced whole body glucose consumption, increased fatty acid oxidation in EDL and soleus, but no alterations in force production of tibialis anterior and soleus muscle [35]. Especially the absence of HF-induced peripheral muscle strength loss [35] is in accordance to our study [6, 33, 34].

All other studies point out that increasing TAC severity is associated with worsening cardiac performance defining the HF phenotype. While in humans HF induces skeletal muscle atrophy [3], we could not observe this effect in mice. Additionally, we and others observed that moderate exercise further deteriorated cardiac function and promoted fiber atrophy in the TAC mouse model.

The data of the current study suggest that TAC in mice in combination with moderate exercise training is not suitable to ameliorate HF associated cardiac dysfunction.

Exercise itself leads to temporarily increased blood pressure, which physiologically can be regulated via peripheral impedance in healthy training individuals. In active muscles, resistance vessels relax to increase blood flow in order to meet the muscles metabolic requirements [38]. Outside the active muscle systemic vascular resistance decreases, which finally leads to an increased heart rate. The LV output is increased due to ensure proper nourishment of the active muscle [38]. In TAC operated mice systolic and diastolic LV filling pressure is significantly increased alongside with an increased vascular stiffness, vascular resistance as well as increased blood pressure and LV filling pressure [39]. These factors fix systemic vascular and LV load at a high level [39]. This condition restricts the capability to regulate blood pressure during exercise [6].

Finally, since physical activity is capable of correcting structural and metabolic alterations in the skeletal muscle of HF patients [4–8], the TAC mouse model especially in combination with exercise intervention does not mirror this.

## Conclusion

TAC is a solid model to study the effects of LV pressure overload in C57BL/6J mice. Our data clearly indicate the suitability of the 27G TAC technique to introduce HF characteristics in mice. Cardiac and skeletal muscle remodeling and function is not ameliorated but rather enhanced by exercise intervention in the 27G TAC mouse model. Other TAC phenotypes (mild/25G or moderate/26G) may affect skeletal muscle structure and performance to a different degree, but this remains unclear.

We observed that TAC alters RV structure in addition to LV remodeling, which indicates development of a biventricular dysfunction. Thus, TAC in mice may be considered as an appropriate model for type 2 PH.

In summary, moderate exercise training intervention in the 27G TAC mouse model is not suitable to study beneficial exercise effects on cardiac remodeling and peripheral cachexia in HF.

## Materials and methods

### Animals, TAC surgery, and sacrifice

**Fig. 4:**
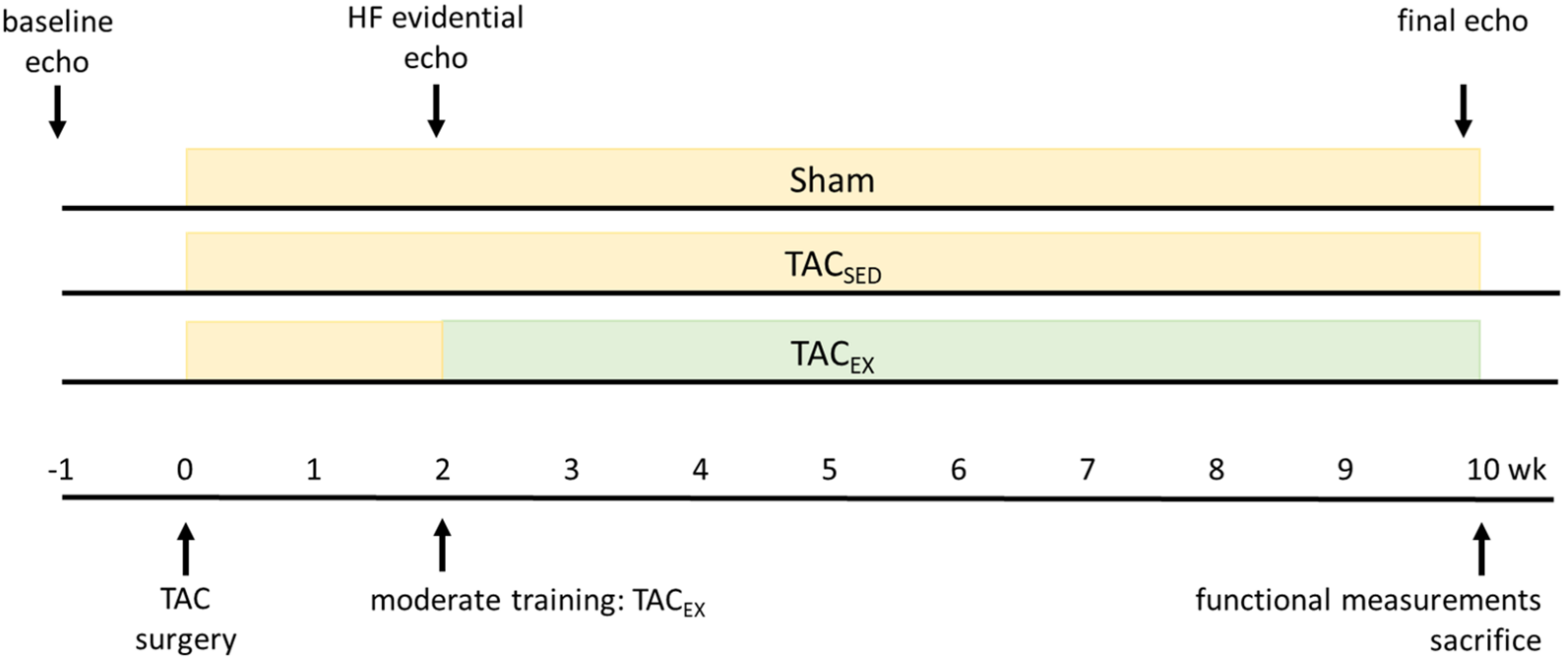
Timeline of study course. *wk* week

All experiments in this study were approved by the local Animal Research Authority, in accordance with the European Directive (TVV 51/18). A total of 45 male C57BL/6J mice (Janvier Labs, Le Genest-Saint-Isle, France) entered the study (see Fig. 4 for study course). The 6 weeks old mice were randomized into two groups: sham-operated control (n = 15) and TAC-operated group (n = 30). A second randomization of the TAC-operated group was done 2 weeks after TAC surgery: TAC + exercise (n = 15), TAC + sedentary (n = 15).

Eight-week old mice weighing 19–24 g, were subcutaneously administered with 5.0 mg/kg carprofene for preoperative analgesia. The mice were sedated by intraperitoneal injection of 0.5 mg/kg Medetomidin, 5 mg/kg Midazolam und 0.05 mg/kg Fentanyl. The chest was opened through a lateral thoracic window from the throat to the second rip. A 27G needle was placed on the transverse aorta and secured using 6.0 prolene to reduce aortic diameter to 0.4 mm. The needle was removed immediately and the chest was closed using 6.0 prolene. The sham-operated control group underwent the same procedure but without binding of the aorta. Post-operative analgesia comprised of 0.1 mg/kb buprenorphine 3× per day, 5.0 mg/kg carprofene 1× per day and additional buprenorphine supply in the drinking water for 2–3 days in total. Mice were weighted and sacrificed 1 day after the last training unit using intraperitoneal injection of 5 mg/kg xylazinhydrochloride, 100 mg/kg ketaminhydrochloride and 0.1 mg/kg atropinsulfate. The hearts were separated, weighted and immediately stored in liquid nitrogen whereas the medial portion of the heart was fixed in 4% paraformaldehyde. Left extensor digitorum longus was removed after ligating it’s proximal tendon using silk braid black, followed by dissecting the muscle without pressure or dragging and finally binding the distal EDL tendon for muscle functional measurement. Muscle preparation was conducted in carbogen (95% O_2_, 5% CO_2_)—flushed Krebs–Henseleit-Buffer (120.5 mM NaCl, 4.8 mM KCl, 1.2 mM MgSO_4_, 1.2 mM NaH_2_PO_4_, 20.4 mM NaHCO_3_, 1.6 mM CaCl_2_, 10 mM dextrose, 1 mM pyruvate, pH = 7.4).

### Echocardiographic measurement of cardiac function in the developing HF

For echocardiography (Vevo 770, Visual Sonics, Canada) mice were anesthetized with 5 vol% isoflurane followed by a 2 vol% isoflurane flow for maintaining anesthesia under continuous ventilation. Baseline measurements were performed at 7 weeks old mice (t = − 1 week) and again at an age of 10 weeks (t = 2 weeks) and 18 weeks (t = 10 weeks). Systolic and diastolic LV thickness, volume, ejection fraction and fractional shortening in control, HF and HF training groups were measured at the indicated time points (see Fig. 4).

### Exercise protocol

The TAC_EX_ group started exercise intervention 2 weeks after TAC operation five times a week for a total of 8 weeks. Maximum VO_2_ max was determined using an exercise tolerance test and a moderate training regime was defined at 60%. According to the previously performed exercise tolerance test to estimate VO_2_max, a training schedule was applied (see Table 3). Treadmill speed was adapted to the group’s slowest individuum regarding the exercise tolerance test. Moderate training was performed in a treadmill at an inclination of 20° including an initial 10 min warm up and final cool down at 40% of the maximum performance and a 40 min training phase at 60% of the maximum performance in between.

**Table 3:** Daily exercise regime of the moderate training group TAC_EX_

	Speed (m/min)
Warm up/40%	10
Training/60%	16
Cool down/40%	10

### Histological evaluation of TAC-induced hypertrophy and fibrosis

Cardiac hypertrophy was evaluated by analyzing cross-sections (3 µm) were stained with hematoxylin and eosin. Area, circumference, as well as the thickness of the left and right ventricular wall were determined using ImageJ (v1.8, NIH, USA). Data are shown in relation to sham. Sirius Red staining was applied for identification and quantification of collagenous fibrotic tissue. For analysis of heart metrics ObjectJ PlugIn (Vischer, N., The Netherlands) was used to measure each ventricle wall 5 times at 5 positions and a polygonal region drawn around the cross section to calculate the heart area and circumference. For fibrosis estimation a threshold for Sirius Red staining intensity was applied and the proportion of interstitial and perivascular fibrosis was calculated in relation to the area of the whole section.

### Muscle function

Left EDL muscle function was determined as described [40]. In brief, EDL was fixed on a force transducer (Aurora Scientific, Aurora, Canada) and equilibrated in an organ bath (Aurora Scientific, Aurora, Canada) at 37 °C in carbogen—flushed Krebs–Henseleit-Buffer for 15 min. To measure EDL force production the muscle was stimulated by an impulse of 700 mA and 0.25 ms with increasing frequencies 1, 15, 30, 50, 80, 120, 150, 300 Hz followed by a one minute rest period using a bipolar high performance stimulator (Aurora Scientific, Aurora, Canada). The tension generated by the EDL muscle contraction is recorded in gram (g) as a function of frequency.

### Slow–fast muscle fiber staining

Serial formalin-fixed and paraffin-embedded EDL and Soleus slices (3 µm) were stained for histological analyses of cross-sectional area. First muscle tissue slices were deparaffinized in 3 × 10 min xylol followed by a descending ethanol series hydration. Antigen retrieval was conducted in target retrieval solution (Dako, Santa Clara, USA). After cooling to room temperature, the slices were washed with deionized water and treated with (20 µg/mL) Proteinase K (Sigma Aldrich, St. Louis, USA) for 10 min at 37 °C, washed with 1× PBS and treated with Peroxidase Block (Dako, Santa Clara, USA) for 10 min at room temperature, washed again with 1× PBS and blocked with serum-free Protein Block (Dako, Santa Clara, USA) for 20 min at room temperature. After another washing step in 1× PBS slices were incubated with an anti-slow skeletal MHC antibody (Abcam, Cambridge, UK) at 4 °C overnight. Slices were washed again in 1× PBS and incubated with a peroxidase-conjugated secondary antibody (Sigma Aldrich, St. Louis, USA) at room temperature for 45 min, washed again with 1× PBS and processed using VECTASTAIN Elite ABC Kit Peroxidase and the DAB Peroxidase Substrate Kit (Vector laboratories, San Diego, USA).

### Data processing and statistics

Heart weight was normalized to body weight, or to tibial length to compensate potential differences in BW.

Statistical analyses were performed using GraphPadPrism6 (San Diego, CA, USA). Data are presented as mean ± standard error of the mean (SEM). Shapiro–Wilks-Test was performed to check for normal distribution. Group differences were analyzed using one-way ANOVA or Dunnett’s multiple comparison. Muscle function (force–time-relationship) was assessed by two-way repeated measures ANOVA. Bonferroni correction was used as post-test. All p-values < 0.05 were considered statistically significant.

## Data Availability

Not applicable.

## Abbreviations

BW: Body weight
CSA: Cross-sectional area
EDL: Extensor digitorum longus
EF: Ejection fraction
ETT: Exercise tolerance test
FS: Fractional shortening
G: Gauge
HF: Heart failure
LCCA: Left common carotid artery
LV: Left ventricle/left ventricular
LVEF: Left ventricular ejection fraction
PH: Pulmonary hypertension
RV: Right ventricle/right ventricular
TAC: Transverse aortic constriction
TAC_EX_: Transverse aortic constriction combined with exercise
TAC_SED_: Transverse aortic constriction combined in sedentary mice
TL: Tibial length
